# The effect of male age on patterns of sexual segregation in Siberian ibex

**DOI:** 10.1038/s41598-018-31463-w

**Published:** 2018-08-30

**Authors:** Muyang Wang, Joana Alves, António Alves da Silva, Weikang Yang, Kathreen E. Ruckstuhl

**Affiliations:** 1 CAS Key Laboratory of Biogeography and Bioresources in Arid Land, Xinjiang Institute of Ecology and Geography, Urumqi, 830011 China; 2 CFE-Centre for Functional Ecology, Department of Life Sciences, University of Coimbra, Coimbra, Portugal; 3 Department of Biological Sciences, University of Calgary, 2500 University Drive Northwest, Calgary, AB T2N 1N4 Canada

## Abstract

Sexual segregation is very common in sexually size dimorphic ungulates and may be the result of different habitat preferences and/or differential social behaviours of males and females. Various hypotheses have been put forward to explain this phenomenon. In the present research, we examined sexual segregation in a quite poorly understood species, the Siberian ibex. The species presents a marked sexual size dimorphism, with adult males weighing double as much as females. We use the Sexual Segregation and Aggregation Statistics (SSAS) to analyze the sex-age patterns of sexual segregation in this species, to understand the relevance of social factors. Our results show that adult Siberian ibex males were socially segregated from females all year round, except during the rutting season. Furthermore, the degree of segregation between females and males was influenced by the age of males. Moreover, the patterns of social segregation within males also increased with male age, reaching maximum values for males of 9 years-old and older, which means male age plays an important role in the sexual segregation of this species. This study clearly shows that social factors play a key role in the sexual segregation of Siberian ibex.

## Introduction

Sexual size dimorphism is considered the main cause of differences in nutritional requirements of mammalian herbivores, both at the inter- and intra-species level^1–3^. Body size dimorphism is also considered to be an important factor for the evolution of sexual segregation at the intraspecific level^4–6^, especially when the sexual size dimorphism is higher than 20%^7^. Sexual segregation has been documented widely in ungulates^8^, in which males and females live in separate groups outside the mating season^5,9^. In terms of defining sexual segregation, it may be described as the differential use of habitat between the sexes (habitat segregation)^10,11^, or as the segregation of males and females into different group types within specific habitats (social segregation)^12,13^. Although some authors consider social segregation as a by-product of habitat segregation^14^, it has been acknowledged that social factors can lead to social segregation independently of habitat segregation^15^. Currently four main hypotheses may provide explanations on the social component of sexual segregation, namely, the social affinity hypothesis (SAH), social factors hypothesis (SFH), activity budget hypothesis (ABH), and reproductive strategy hypothesis (RSH).

The social affinity hypothesis (also referred to as ‘social preference’ hypothesis)^16,17^ proposes that (1) males and females differ in their ontogenetic behaviour, i.e., different social motivation that could result in preference for the same sex outside the breeding season^17^. This hypothesis also predicts that young males, which are sexually inactive, show marked levels of same-peer interaction from an early stage in their life^17^, and if these preferences extend to adulthood, they might lead to “autosegregation”^18^. (2) Females can more efficiently develop skills associated with the rearing of offspring in female-only groups, while males may more efficiently develop fighting skills, evaluate rivals and establish dominance hierarchies in male-only groups. Therefore, the formation of single-sex groups may be a functional outcome of the individual’s preferences for same-sex peers^19^. Regarding younger males, they will not join male-only groups of older males due to differences in body size and fighting skills^19^. According to this hypothesis, the sexual incompatibility may be accentuated as males age and develop secondary sexual characteristics^19,20^. Therefore, social segregation is not only expected based on sex, but also age class^21^.

In addition, SAH also proposes that social segregation between sexes is due to females avoiding males of all size classes to minimize any form of social threat to their newborns, either when they engage in agonistic acts^17,22^, or because male-male aggression affects female group cohesion^23^. Furthermore, females close to parturition or with newborns, become more aggressive^24^, which would result in sexual segregation but also the segregation of females at different reproductive stages.

The social factors hypothesis (SFH) predicts the occurrence of single-sex groups based on the importance of social interaction to increase fighting skills and social learning, to avoid costly social interactions in the presence of the opposite sex, and ultimately to increase the reproductive success of males (reviewed by Main *et al*.)^25^. Therefore, this hypothesis emphasizes that behavioural compatibility is key to enhancing group cohesion, while incompatibility would result in social segregation outside the mating season^18^. According to the SFH, a mutual sexual avoidance is expected outside the rutting season, due to aggressive behaviour, which associated with intersexual affinity and social preferences will lead to unisex groups^25^.

Connecting foraging demands and group cohesion, the activity budget hypothesis (ABH) proposes that segregation arises from differences in time spent active or inactive by females and males due to differences in their body size^26,27^. This hypothesis states two aspects: the smaller females are less efficient at digesting forage than bigger males (20+ % bigger), and therefore compensate their lower digestive efficiency by foraging longer than males. Males, on the other hand, will spend more time ruminating or lying to digest forage. As a consequence, individuals that differ in body size, are more likely to segregate socially as their members will be less behaviourally synchronized^27,28^. This apparent asynchrony in behaviour and potentially costly synchrony between the sexes leads to animals with similar activity budgets to form their own groups and segregate from other types of groups^29–31^. The ABH thus predicts that similar-sized individuals will tend to aggregate, while mixed-body size groups will tend to break up^26^.

The reproductive strategy hypothesis^32^ states that sexual segregation results from sexual differences in the reproductive tactics used to maximize their reproductive success^9,32,33^. According to RSH, during the birthing and lactation seasons (outside the rut) sexual habitat segregation should be stronger, particularly for females with vulnerable offspring^25^. This may result from differential habitat requirements or energetic needs^25,33^, associated with different reproductive strategies^34^: Females with offspring will select safer habitats, even at the expense of food quality, to ensure the survival of the newborns. Males will select habitats with higher quality food, even at the expense of a higher predation risk, as a strategy to increase their physical condition and ensure reproductive success^25,33,34^.

Although most of these hypotheses are concerning sexual segregation between adult males and females, age can also affect social segregation, and considering age can also help to understand some aspects of this widespread phenomenon^29,35^. In fact, age will have repercussions both on energetic demands and social interactions, which can lead to a same-sex age segregation, on top of the commonly observed sexual segregation. The potential effects of age on sexual segregation patterns have been rarely studied^36,37^, and even fewer have tested how male age influences sexual segregation^19,38^.

In this paper, we investigated the sexual and age segregation of Siberian ibex (*Capra sibirica*) in the central Tianshan Mountains, China. The Siberian ibex is a sexually dimorphic species, with females reaching their maximal growth around 3 years old, while males continue growing until 9 years old. Although, males have a slower growth rate than females, the sexual body size dimorphism becomes larger than 20% hen males become 3 or 4 old^39,40^. In adulthood, males can be up to two times larger and heavier than females^39^. The rutting season of Siberian ibex in the central Tianshan Mountains usually starts in October and ends in December, and the birthing season occurs in May^39^. Most ibex stay in mixed-sex groups during the rut season and split into single-sex groups after the rut^39^. Using direct observations, we aim to analyze the monthly patterns of sexual and age segregation in this species. Based on the previously described sexual segregation hypotheses, and their associated predictions, we expect that (1) sexual segregation will occur all year around except during the rut season, (2) sexual segregation should be stronger during birthing and lactation, (3) the degree of sexual segregation will increase with male age, (4) inter-male segregation will increase with male age.

## Results

### Proportion of mixed-sex groups among all observed social groups

The proportion of mixed-sex groups observed changed significantly over the months. Monthly fluctuations of the proportion of mixed-sex groups were higher from November to March, with a peak in December, and with smaller proportions in summer (Table 1). The presence of mixed-sex groups was more frequent in December, than in any other month. Solitary females were mostly found from May to July, and also in December, while solitary males were mostly observed from December to February (Table 1).

**Table 1:** Monthly fluctuations of each group type observed. Sample size is given in brackets.

Month	Male group (%)	Female group (%)	Mixed-sex group (%)	Solitary female (%)	Solitary male (%)
January	7.9 (17)	53.7 (115)	38.3 (82)	3.3 (7)	6.1 (13)
February	13.2 (23)	47.1 (82)	39.7 (69)	2.3 (4)	8.1 (14)
March	10.7 (16)	63.8 (95)	25.5 (38)	1.3 (2)	3.4 (5)
April	16.6 (33)	64.3 (128)	19.1 (38)	0.5 (1)	3.0 (6)
May	18.4 (36)	70.9 (139)	10.7 (21)	9.2 (18)	1.0 (2)
June	20.8 (25)	77.5 (93)	1.7 (2)	5.8 (7)	0.8 (1)
July	13.9 (9)	83.1 (54)	3.1 (2)	6.2 (4)	0 (0)
August	23.2 (16)	71.0 (49)	5.8 (4)	2.9 (2)	1.5 (1)
September	19.8 (20)	74.3 (75)	5.9 (6)	3.0 (3)	1.0 (1)
October	7.5 (8)	75.7 (81)	16.8 (18)	0.9 (1)	0 (0)
November	17.1 (25)	54.1 (79)	28.8 (42)	3.4 (5)	2.1 (3)
December	10.6 (19)	32.2 (58)	57.2 (103)	6.7 (12)	7.2 (13)

### Intersexual Segregation in Relation to Male Age

**Figure 1:**
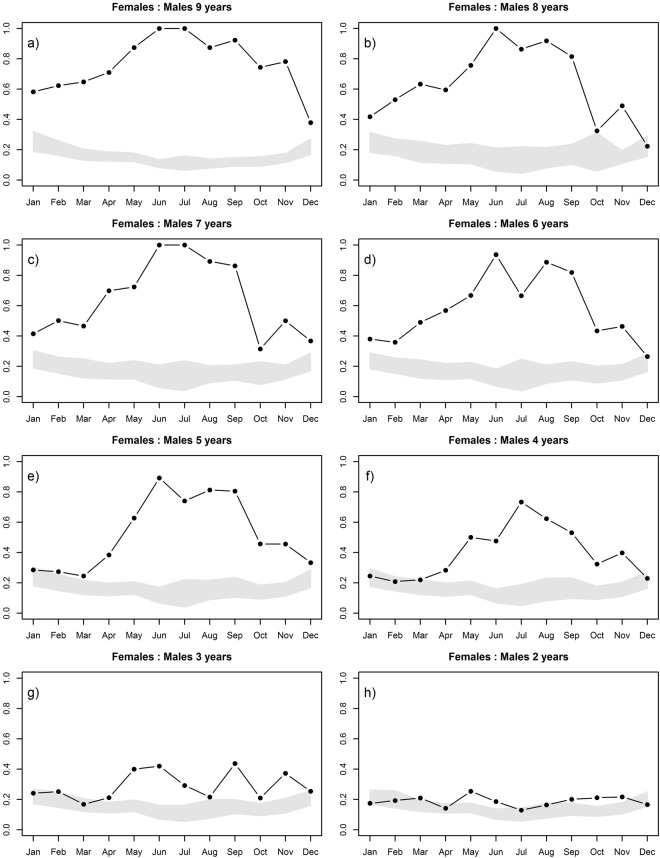
Monthly patterns of sexual segregation and aggregation between females and males of different ages (i.e. (**a**) 9 year old males, (**b**) 8 year old males, (**c**) 7 year old males, (**d**) 6 year old males, (**e**) 5 year old males, (**f**) 4 year old males, (**g**) 3 year old males, (**h**) 2 year old males, respectively) in Siberian ibex. The SSAS indicates significant sexual segregation or aggregation when the observed value (black point) falls above or below the SSAS expected interval (grey area), respectively.

Adult male and female Siberian ibex were sexually segregated all year round, except during the peak of the rutting season (December), when adult males of different ages and females sometimes were randomly associated (Fig. 1). The SSAS values increased with male age, reaching values closer to or equal to 1 in the summer months (Fig. 1). During these months, and considering the low proportion of solitary males and females observed (Table 1), females and adult males (6 years and older) prefer to aggregate with same-sex peers.

Females and 2-year old males were randomly associated for most of the year (Fig. 1h). Three to 5-year-old males were segregated from females from April to November, meaning that subadult males occurred more frequently in single sex groups than in mix-sex groups during these months (Fig. 1e–g), and they were randomly associated with females during the winter months (November to March) (Fig. 1e–h).

### Age-related Inter-Male Segregation

**Figure 2:**
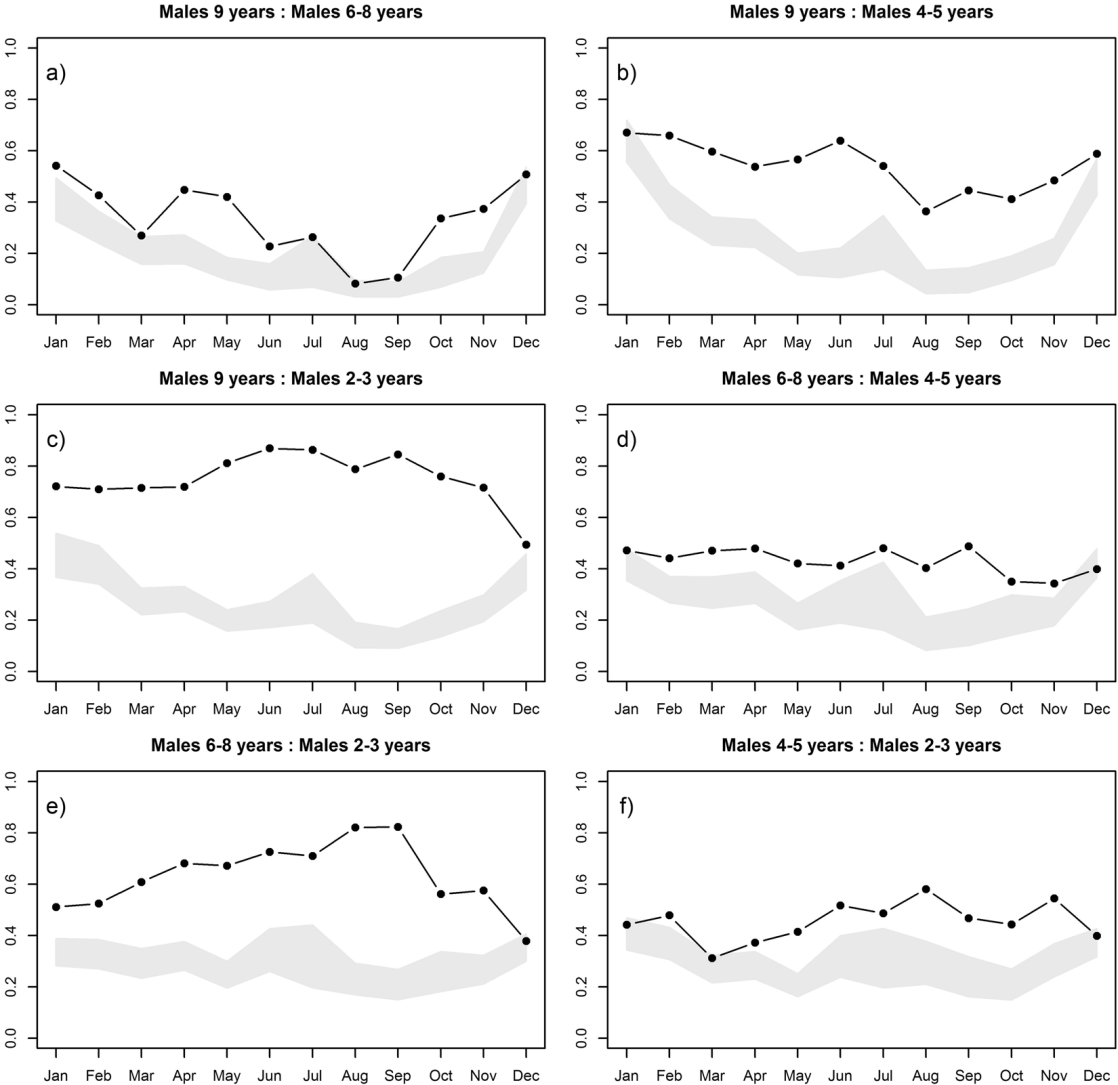
The monthly patterns of age class related segregation and aggregation for male Siberian ibex, comparing: (**a**) ≥9 and 6–8 year olds; (**b**) ≥9 and 4–5 year olds; (**c**) ≥9 and 2–3 year olds; (**d**) 6–8 and 4–5 year olds; (**e**) 6–8 and 2–3 year olds; (**f**) 4–5 and 2–3 year olds. The SSAS indicates significant segregation or aggregation when the observed value (black point) falls above or below the SSAS expected interval (grey area), respectively.

When we analyzed segregation among males of different age classes, we found an age-related segregation during most of the year, with males grouping more often with other males of similar age-classes (Fig. 2). These results indicate that Siberian ibex males show age-related segregation, with males being more aggregated with other males of similar age, and completely segregated from males of different ages (Appendix 1).

## Discussion

With a high degree of sexual body size dimorphism and in agreement with what we predicted, Siberian ibex showed remarkable monthly sexual segregation. Adult males and females segregated all year around except in the rut season, when female and male ibex spent much more time in mixed-sex groups for mating. The segregation between adult males and females peaked in the summer season, in which mixed-sex groups were rarely seen. However, it is also important to notice that adult females also segregated from 4- and 5-year-old males (in particular during spring and summer), with whom they share a lower degree of sexual body-size dimorphism (but already higher than 20%).

Contrary to what we expected, our results indicated that adult male and female Siberian ibex were even segregated during the rutting season, and only showed a tendency to randomly associate at the peak of the rut (December). Similar results have been reported for Alpine ibex (*Capra ibex*), in which there was a higher sexual segregation during the rutting period than expected^19^. It appears that Siberian ibex and Alpine ibex have a similar promiscuous mating system, i.e., they group together freely^19^, which explains the low segregation index during the rutting period in our Siberian ibex study.

There was a clear increase of sexual segregation in our Siberian ibex as male age increased. As the males grow older than six years, sexual segregation between the sexes becomes stronger than between females and males younger than 5 years old. Increasing sexual segregation with male age has also been found in other species (*Capra ibex*, *Cervus elaphus*)^19,38^. In *Ovis aries*, segregation between females and their young even started when males were 3 weeks of age, which provides new arguments in support of the social affinity hypothesis in ungulates^41^. Subadult Siberian ibex were randomly associated with adult females during some months of the year, but this was even more evident for the younger males (2 and 3 years old). This result can be explained by low levels of pseudosexual play exhibited by subadult males, making them more tolerable in matriarchal groups outside the rutting season. Weckerly (2001) found that the large Roosevelt elk (*Cervus elaphus roosvelti*) males are more aggressive than younger males, and thus suggested that the big males’ asocial behaviour would lead them to either be solitary or to segregate from females and younger males into small groups of older males outside the breeding season^42^.

The ‘social affinity’ hypothesis (SAH) predicts that sexual segregation increases with male age, because males most likely change their social motivations and show more behavioural incompatibility with their mothers or other females as they grow up^43^. This hypothesis has been supported in studies on Alpine ibex^44^ and Nubian ibex (*Capra ibex nubiana*)^45^, and it is also supported by our own results for Siberian ibex. Sexual segregation in our population peaked from April to September, which corresponds to the birthing and rearing season. During this period, pregnant females opt to leave the group and move to predator-safe habitats for giving birth and lactating, as predicted by the reproductive strategy hypothesis^33,34^. Alternatively, it has been suggested that females close to parturition may become aggressive to any adults and might isolate to strengthen the mother-kid bond^19,46^. Habitat and social factors seem to play an important role in promoting sexual segregation during this period^38^.

Our results confirmed that age segregation of Siberian ibex males increased as the difference in age increased. Moreover, males were seen more often in male groups of similar age, than with males of different ages or even with females. Behavioural and social mechanisms were suggested as the main factors resulting in males of different ages to live in separate groups for some species^19,38,47,48^. Both the ‘social affinity hypothesis’, which states that individuals will group with same age-sex peers due to similar social needs and constraints^18,19^, and the ‘activity budget hypothesis’, stating that sexual dimorphism in body size and energetic demands would lead to single-sex and age-group formation^35,49^, could explain our observed age-related inter-male segregation in Siberian ibex. For example, differences in activity budgets of bighorn sheep (*Ovis canadensis*) were found between male age classes^26^ and similar age-sex bighorn sheep had higher behavioural synchrony^29^, which resulted in individuals of similar size to form groups of their own, segregating socially from the others. Both activity budgets and social factors may lead to sexual segregation in *Capra hircus*, and thus activity budget hypothesis seems to be capable of explaining the results found^50^. However, the activity budgets hypothesis was not enough to explain the sexual segregation found in some species^50–53^. These studies clearly indicate that there is not a strong association between behavioral synchronization and segregation, and that differences in activity budgets and synchrony alone are insufficient to explain social segregation in these species.

Although we cannot distinguish which hypothesis (social affinity or activity budget) may better explain the observed inter-male segregation in Siberian ibex, it is clear that as males grow older, their segregation from females and younger males becomes stronger. If the activity budget hypothesis could explain the sexual segregation of Siberian ibex, subadult males should form their own groups (subadult males only) and socially segregate from adult males and females. Surprisingly, very few subadult male groups were found in our study. In bighorn sheep, subadults altered their own optimal time budget to synchronize with adult individuals in the group (likely to maintain group cohesion), whether they associated with nursery groups or bachelor groups^26,29^. However, Ruckstuhl and Festa-Bianchet found that as subadult bighorn ram numbers increased, they formed same-age groups^35^. Combined with the results of subadult male Siberian ibex’ random association with females, and segregation from adult males, we conclude that the segregation between females and subadult males from April to June is likely a by-product of the birthing and lactation seasons, supporting RSH. This was the only period in which groups composed only of subadult males were observed. Females prefer to isolate into safer habitats to give birth, which can lead to social and habitat segregation during that specific season, as previously shown for other ungulate species^5,25,38,54^.

The random association, instead of a clear aggregation, found between adult males and females during the rut may also be related to the temporal scale used in our sampling survey. In this case, if sexual aggregation occurs at a smaller temporal scale (probably one or two weeks), is possible that our monthly sampling may not have been enough to capture such short aggregation pattern. In fact, Alves *et al*. reported that a 15-day-long survey may be needed to observe a significant aggregation between the sexes during the breeding period in red deer^38^. In Alpine ibex, older males segregate socially from females soon after the rut, while subadult males segregate later^42^, which is in concordance with our results for Siberian ibex. This, associated with the promiscuous mating system of Siberian ibex may be the reason for the lack of clear aggregation between adult males and females found in our study. In Alpine ibex, old and young males use different reproductive tactics. Old males use a ‘tending’ tactic, in which they stay close to females, while younger males use a sneaking tactic ‘coursing’, and generally are further away from females, with little opportunity to access receptive females^55^. Therefore, one possible explanation of the above phenomenon is that young males stay further away from those mating groups for most of the time (distances are greater than 50 m), but in the proximity of the group.

Another possible explanation of why young males stay with females for a longer period after the rut may be related with females going through a second estrus during one reproductive period, if impregnation and the development of the corpus luteum do not occur during the first estrus^56^. Therefore, younger males, who are expelled from the mating groups by older males in the peak of the rutting season, have another chance to mate those females after the peak of the rutting season^39^. Younger males started to rejoin females after the older males left, which resulted in the observed random association between subadult males and females (from January to March) in our study.

Sexual segregation of Siberian ibex was found for most of the year. The degree of sexual segregation seems to increase with male age, which may be related to social motivations or with the increase of sexual body-size dimorphism and consequently, different energetic demands. The male age class segregation found in our study supports the idea that different energetic needs, social motivations, or habitat requirement are not only important factors implicated in inter- but also intra- sexual segregation of Siberian ibex. Our results provide support for hypotheses related to social segregation, like the social affinity and the activity budget hypotheses, but can for some time of the year also be explained by hypotheses inherent to habitat segregation (the reproductive strategy and forage-selection hypotheses)^57^, which was found during the birthing-lactating season. Due to these sexual and age segregation patterns, we conclude that social factors seem to play a key role in sexual segregation of Siberian ibex, for most of the year. Further research should concentrate on disentangling different types, causes and mechanisms that are involved in social segregation.

## Materials and Methods

### Ethical Approval

No further approval by an Ethics Committee was required, as behavioural observations at a distance in this study were non-invasive.

### Study area

The study was conducted in the Tengger Mountain Range of Eastern Tianshan, Xinjiang, China (N 43°13′–N 43°43′, E 86°30′–E 87°29′). Our total study area was around 1700 km², with rugged ridges and narrow valleys and elevations of 1450 to 4479 m a.s.l. This region has a semi-humid to semi-arid transition zone with a temperate continental climate, and a high altitude, resulting in a cold and arid climate^58^. The annual average precipitation is 663.4 mm and annual average frost – free period of about 150 days^59^. The annual average temperature is −1.0 °C. Extreme high temperatures of up to +30.5 °C are typical in July, and extreme low temperatures down to −30.2 °C are common in January. In this area, the habitat is dominated by coniferous forests under the tree line (2100 m altitude), and by alpine grasslands and bare rocks above the tree line. *Cyperaceae* and *Poaceae* are relatively dominant in the local plant community, with an admixture of other families, such as *Polygonaceae*, *Asteraceae*, *Papilionaceae*, *Ranunculaceae*, and *Rosaceae*. Siberian ibex and red deer (*Cervus elaphus*) are common ungulates in the study area. Carnivores, such as snow leopards (*Uncia uncia*), wolves (*Canis lupus*), and raptors, such as cinereous vultures (*Aegypius monachus*), Lammergeiers (*Gypaetus barbatus*) and golden eagles (*Aquila chrysaetos*) are present. A large number of domestic sheep and goats from the town of Saerdaban stay in our study area from June to October each year.

### Data collection

Direct observations of ibex were made once a month from 2013 to 2015. We conducted our survey by car at a low speed (less than 20 km /h) or on foot, whenever necessary. The length of transects varied from 7 to 20 km. In each month, we surveyed Siberian ibex along the same transect to make sure the sampling was balanced. We stopped every 2–3 km along each transect line and searched for ibex using binoculars (magnification 8×) and a telescope (magnification 20–60×). The survey was conducted from sunrise to sunset to cover the entire daytime. In total, we collected data on 1723 groups during the surveys, including 214 groups in January, 174 groups in February, 149 groups in March, 199 groups in April, 196 groups in May, 120 groups in June, 65 groups in July, 72 groups in August, 101 groups in September, 107 groups in October, 146 groups in November, and 180 groups in December.

In most cases, we were able to observe Siberian ibex within a distance of 300 m. At this distance, most males could be aged by counting horn annuli, following Bon *et al*.^19^. For all groups observed, the sex, age classes, and group compositions were recorded. For each observed group, we counted all females (adult or subadult) and determined the age of all males by counting horn annuli, when visible. Siberian ibex males at 6 years old start their participation in the rut, and males younger than 6 years old do not compete over mating. Therefore, only males aged 6 years or older were defined as adults^39,60^. Moreover, since males continue growing until they are 9 years old^39,61^, we further divided the adult males into two age-classes: 6 to 8-year-olds, and males of ≥9 years. In total, we had the following age-sex classes: adult females ≥1-year-old; and males 2–3 years old, males 4–5 years old, males 6–8 years old, and males ≥9 years old. A group was defined as inter-individual distances not exceeding 50 meters, which is a fairly large distance and nearest neighbor distances were typically much closer (the distance between individuals was visually estimated in ibex body lengths)^62^.

### Data analysis

#### Sexual segregation

The sexual segregation and aggregation statistics (SSAS), proves to be a useful tool to quantify sexual segregation^63^, and was used to test for segregation and aggregation in our study ibex according to the following equation:

$${\rm{SSAS}}=1-\frac{N}{X\times Y}\times {\sum }_{i=1}^{k}\frac{{X}_{i}\times {Y}_{i}}{{N}_{i}}$$

where N = X + Y, X is the total number of animals of class I sampled, Y is the total number of animals of class II sampled, k the total number of groups. X_i_ is the number of animals of class I in the ith group, Y_i_ is the number of animals of class II in the ith group and N_i_ = X_i_ + Y_i_. The SSAS allows one to test for the null hypothesis of a random association between the age-sex classes (e.g. males and females, older and younger males) against the two alternative hypotheses: segregation (i.e. the sex ratio of the groups is strongly deviating from the sex ratio of the population) and aggregation (i.e. the group sex ratio is similar to the population sex ratio)^37^. When animals mainly occur in mixed age or sex groups, the SSAS is lower than expected and close to 0, otherwise, the SSAS index is higher than expected and close to 1 when animals appear mostly in unisex groups. SSAS indices vary between 0 (complete aggregation) and 1 (complete segregation) and needs to be interpreted according to the confidence interval of the expected values.

Moreover, we also performed SSAS tests to compare segregation and aggregation of each age and sex class combination. For a given month, the data from the years were pooled, as the sexual segregation patterns did not differ between years (χ^2^_2_ = 3.819; P = 0.148). SSAS was calculated using R 3.3.1^64^.

## Electronic supplementary material

Appendix

## Data Availability

The datasets generated and/or analyzed during the current study are available from the corresponding author on reasonable request.
