# Supplementary material for: The effect of male age on patterns of sexual segregation in Siberian ibex
Source: Sci Rep. 2018 Aug 30;8:13095. doi: 10.1038/s41598-018-31463-w (PMC6117266; doi:10.1038/s41598-018-31463-w)
Supplement: Supplementary file 1 — Appendix [file 41598_2018_31463_MOESM1_ESM.pdf]

The effect of male's age on patterns of sexual segregation in Siberian ibex

Muyang Wang1, Joana Alves2, António Alves da Silva2, Weikang Yang1*, Kathreen E. Ruckstuhl3

1 Key Laboratory of Biogeography and Bioresources in Arid Land, Xinjiang Institute of Ecology and Geography, Chinese Academy of Sciences, Urumqi, 830011 China; 2 CFE-Centre for Functional Ecology, Department of Life Sciences, University of Coimbra, Portugal; 3 Department of Biological Sciences, University of Calgary, 2500 University Drive Northwest, Calgary, AB T2N 1N4, Canada

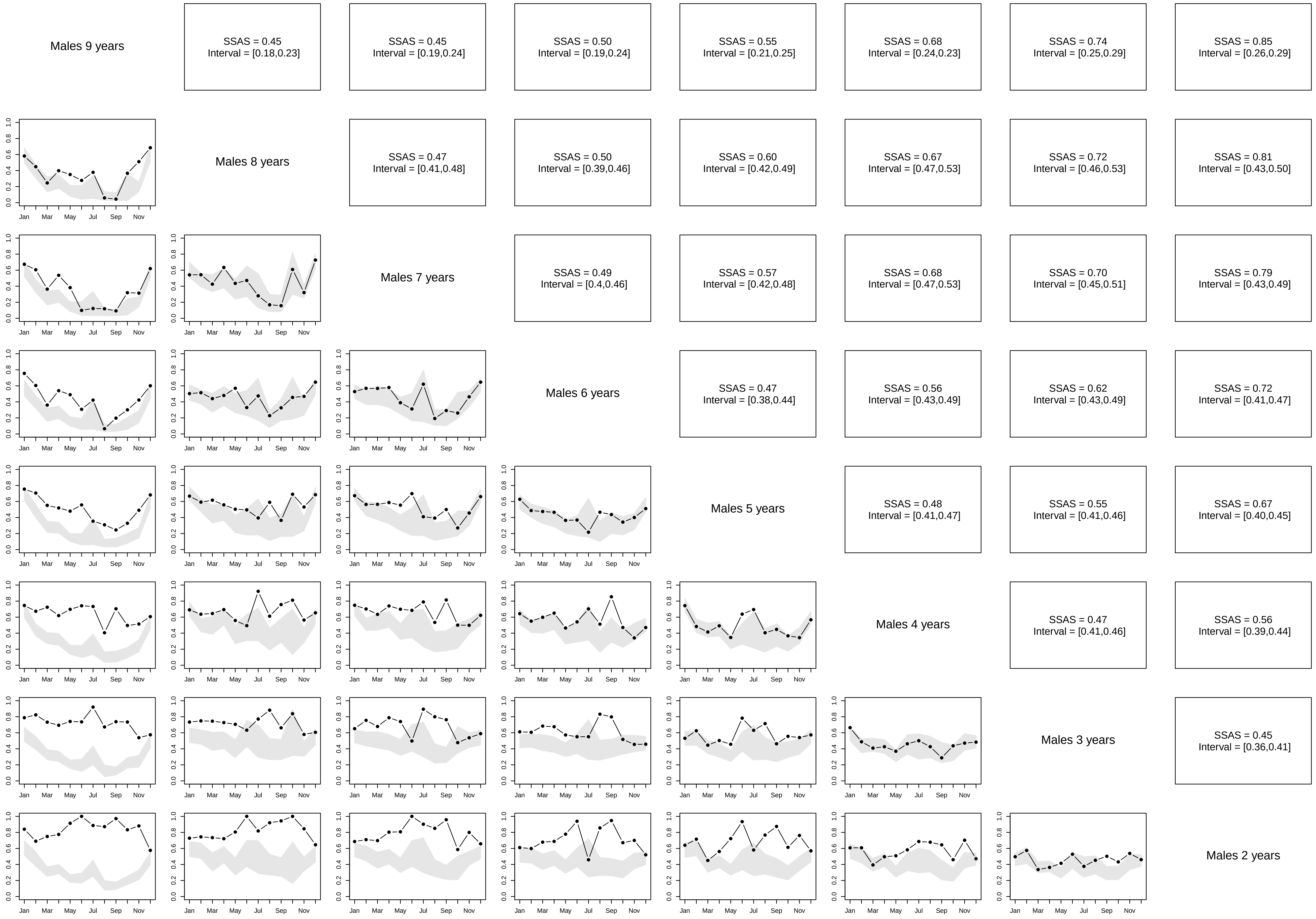
Males 9 years
SSAS = 0.45
Interval = [0.18,0.23]
SSAS = 0.45
Interval = [0.19,0.24]
SSAS = 0.50
Interval = [0.19,0.24]
SSAS = 0.55
Interval = [0.21,0.25]
SSAS = 0.68
Interval = [0.24,0.23]
SSAS = 0.74
Interval = [0.25,0.29]
SSAS = 0.85
Interval = [0.26,0.29]
Males 8 years
SSAS = 0.47
Interval = [0.41,0.48]
SSAS = 0.50
Interval = [0.39,0.46]
SSAS = 0.60
Interval = [0.42,0.49]
SSAS = 0.67
Interval = [0.47,0.53]
SSAS = 0.72
Interval = [0.46,0.53]
SSAS = 0.81
Interval = [0.43,0.50]
Males 7 years
SSAS = 0.49
Interval = [0.4,0.46]
SSAS = 0.57
Interval = [0.42,0.48]
SSAS = 0.68
Interval = [0.47,0.53]
SSAS = 0.70
Interval = [0.45,0.51]
SSAS = 0.79
Interval = [0.43,0.49]
Males 6 years
SSAS = 0.47
Interval = [0.38,0.44]
SSAS = 0.56
Interval = [0.43,0.49]
SSAS = 0.62
Interval = [0.43,0.49]
SSAS = 0.72
Interval = [0.41,0.47]
Males 5 years
SSAS = 0.48
Interval = [0.41,0.47]
SSAS = 0.55
Interval = [0.41,0.46]
SSAS = 0.67
Interval = [0.40,0.45]
Males 4 years
SSAS = 0.47
Interval = [0.41,0.46]
SSAS = 0.56
Interval = [0.39,0.44]
Males 3 years
SSAS = 0.45
Interval = [0.36,0.41]
Males 2 years
